# Grapevine gray mold disease: infection, defense and management

**DOI:** 10.1093/hr/uhae182

**Published:** 2024-07-10

**Authors:** Mati Ur Rahman, Xia Liu, Xiping Wang, Ben Fan

**Affiliations:** Co-Innovation Center for Sustainable Forestry in Southern China, Department of Forest Protection, College of Forestry and Grassland, Nanjing Forestry University, Nanjing 210073, China; Co-Innovation Center for Sustainable Forestry in Southern China, Department of Forest Protection, College of Forestry and Grassland, Nanjing Forestry University, Nanjing 210073, China; State Key Laboratory of Crop Stress Biology in Arid Areas, College of Horticulture, Northwest A&F University, 712100 Yangling, Xianyang, Shaanxi, China; Co-Innovation Center for Sustainable Forestry in Southern China, Department of Forest Protection, College of Forestry and Grassland, Nanjing Forestry University, Nanjing 210073, China

## Abstract

Grapevine (*Vitis vinifera* L.,) is among the world’s leading fruit crops. The production of grapes is severely affected by many diseases including gray mold, caused by the necrotrophic fungus *Botrytis cinerea*. Although all *Vitis* species can be hosts for *B. cinerea*, *V. vinifera* are particularly susceptible. Accordingly, this disease poses a significant threat to the grape industry and causes substantial economic losses. Development of resistant *V. vinifera* cultivars has progressed from incidental selection by farmers, to targeted selection through the use of statistics and experimental design, to the employment of genetic and genomic data. Emerging technologies such as marker-assisted selection and genetic engineering have facilitated the development of cultivars that possess resistance to *B. cinerea*. A promising method involves using the CRISPR/Cas9 system to induce targeted mutagenesis and develop genetically modified non-transgenic crops. Hence, scientists are now engaged in the active pursuit of identifying genes associated with susceptibility and resistance. This review focuses on the known mechanisms of interaction between the *B. cinerea* pathogen and its grapevine host. It also explores innate immune systems that have evolved in *V. vinifera*, with the objective of facilitating the rapid development of resistant grapevine cultivars.

## Introduction

Grapevines (*Vitis vinifera* L. and related *Vitis* species) are among the most widely cultivated and commercially vital horticultural fruit crops in the world. Cultivars of *V. vinifera* cultivars used in the production of wine and table grapes are generally highly susceptible to diseases caused by bacterial viral, oomycetes and fungal pathogens and grape quantity and quality have shown significant declines in recent years. Among the most important grapevine fungal diseases worldwide are gray mold [1, 2], downy mildew [3], powdery mildew [3], and black rot [4].

Gray mold (also called botrytis bunch rot), caused by the necrotrophic fungus *Botrytis cinerea*, is responsible for significant recent decline in the quantity and quality of wine grape harvests [5, 6]. Most European grapevine cultivars show some susceptibility to gray mold [7–10]. Gray mold can cause yield losses ranging from 20–50%, mostly attributed to the decay of mature berries in the field or storage [11]. According to reports, Australia’s yearly revenue loss from botrytis infections in wine and grapes was estimated to be AUS$52 million [12]. *Botrytis* disease was predicted to cause a US$10 billion to US$100 billion yearly loss on a worldwide scale [13, 14]. To this purpose, an estimated US$40 billion in economic losses are seen yearly in the United States alone in order to control the roughly 30%–40% post-cultivation losses caused by *Botrytis* infections [15].

The pathogen thrives throughout the grapevine vegetative cycle in conditions of high relative humidity and warm temperatures [16]. After infection, *B. cinerea* undergoes a brief biotrophic phase within the plant before switching to a necrotrophic phase. *B. cinerea* has the ability to infect a diverse array of plant species, with the total surpassing 1400 [17]. *B. cinerea* flourishes in vineyard soil, especially on decaying plant debris, as part of the microflora [18]. During fruit ripening the disease pathogen infiltrates the fruits, resulting in the development of necrotic lesions accompanied by substantial fungal proliferation [19]. The infection of berries often begins with the introduction of airborne conidia from sources that have survived the winter [20, 21]. When *B. cinerea* comes into contact with plant tissue, it releases phytotoxins that promote cell death and enzymes that break down the cell wall. Furthermore, the pathogen can alter the host’s metabolism in order to facilitate colonization [22].

Breeding programs using generative hybridization have been established since the 19th century [23]. Grapevine species originating from East Asia and North America exhibit remarkable levels of resistance to pathogens [23, 24]. The wild species mentioned in this review are often used as rootstocks in breeding programs due to their high compatibility with *V. vinifera* [25]. This compatibility allows for effective hybridization, resulting in progeny that exhibit resilience to both abiotic and biotic stressors [23, 26, 27]. The breeding of grapes, on the other hand, is a laborious task that requires a significant amount of time. For instance, complete resistance to powdery mildew required six successive crossings between *V. vinifera* and *Muscadinia rotundifolia* [28].

The quest to develop resistant variants has spurred significant efforts among scientists, leading them to explore novel breeding approaches to understand the genetic basis of biotic resistance in the grape genome. One potential strategy is to combine classical selection with marker-assisted selection (MAS), which can introduce specific loci into the resistant lines [29, 30]. Several countries have undertaken various breeding programs with the aim of obtaining cultivars with both disease resistance and high-quality berries [31]. The use of molecular markers facilitates the acceleration and cost reduction of the selection process, while ensuring the development of cultivars with novel gene combinations, a feat that is almost unattainable with conventional breeding techniques [32, 33].

Genetic engineering methods are used in the second strategy to alter/edit the genome. This study includes a range of techniques aimed at deliberately modifying the genetic makeup of plants, specifically targeting grape varieties, with the ultimate goal of developing resistant varieties that possess the desirable traits sought by both growers and consumers [34, 35]. The CRISPR/Cas9 gene editing approach, a promising tool discovered in recent years, is used to genetically modify grapes in order to coupe resistance against fungal diseases [36, 37]. CRISPR/Cas9 may modify genes, suppress/activate gene expression, regulate gene function and modify epigenomes. CRISPR/Cas9-targeted mutagenesis creates non-transgenic modified plants with stable mutations that can be passed down across generations. The production of genetically modified non-transgenic organisms is not explicitly prohibited by the law in some countries. The application of CRISPR/Cas9 editing has significant potential to improve the resilience of grape varieties. Researchers have conducted a study to identify novel target genes in grapevines that can be edited using CRISPR/Cas9 technology to improve resistance to infection [35, 38].

To date, the genetic sequences of the domesticated grape variety *V. vinifera* [39, 40], its wild counterpart *V. vinifera* sylvestris [41–43], and the fungal pathogen *B. cinerea* have been successfully determined [44]. The identification of gene systems linked to resistance or susceptibility to widespread diseases like gray mold has been made easier by recent genetic study on grapes [45–47]. Our article summarizes the efforts made to explore the disease resistance mechanism of *B. cinerea* (i.e., identification of relevant genes and pathways, gene and genome editing). Furthermore, we highlight the research gaps and provide guidelines for minimizing the losses caused by this disease.

## Pathogenesis of *B. cinerea* in grapevine

The fungal taxon known as *Botrytis*, belonging to the family *Sclerotiniaceae*, is considered to be one of the most extensively studied and ancient genera. Based on current taxonomic investigations, it has been shown that there are more than 35 distinct species of *Botrytis*. Among these species, *B. cinerea* has received considerable attention and has been extensively studied [48, 49]. Contrary to the common necrotrophic plant diseases caused by the genus Botrytis, *B. cinerea* has a broader host range as a generalist pathogen, allowing it to infect multiple fruit plant species (i.e., strawberries [50], apple [51], blueberry [52], kiwifruit [53], Arabidopsis [54], and including major agricultural crops [49]). *B. cinerea* has the ability to infect both undamaged leaves and stems but tends to preferentially target softer tissues such as fruits, vegetables, and flowers, particularly under optimal conditions. Its detrimental effects are manifested in significant reductions in crop yield, which occur both before and after harvest [55]. *B. cinerea* infection is thought to be facilitated by the widespread release of plant cell wall degrading enzymes (PCWDEs) and non-selective toxins [55, 56]. Nevertheless, new research has shown that this fungus has a more diverse range of capabilities than originally thought. Current understanding, although limited, suggests that the infection caused by *B. cinerea* is a complex phenomenon influenced by the interaction of multiple variables that together contribute to the progression and intensity of the disease [55].

**Figure 1:**
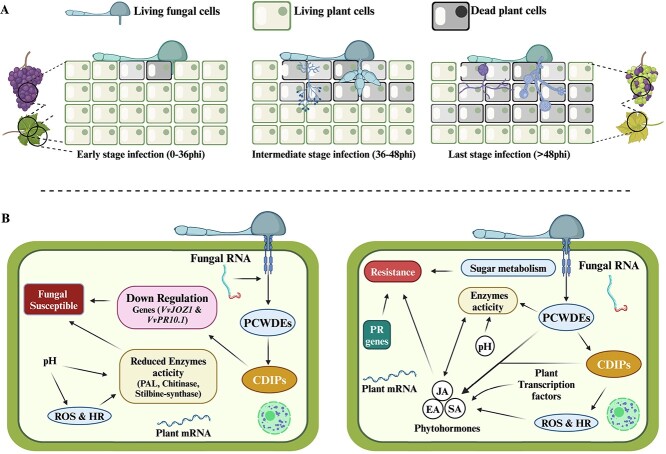
Systematic steps involved in *Botrytis cinerea* infection of grapes. **A** The illustration explains the chronological progression of developmental processes that occur during the three primary phases of *B. cinerea* infection. It is important to note that this diagram may vary significantly depending on the circumstances. During the early 0–24 hours post inoculation (hpi), spores undergo germination and develop early infection spots with the assistance of appressoria and infection cushions (ICs). The fungus usually lives on the host’s surface and uses cell death-inducing proteins (CDIPs) to kill a few host cells. Depending on the host and environment, the hyphae enter host tissues and destroy more plant cells 24–48 hours post-infection (see Fig. 2 for detail). However, the fungus also faces a counterattack from plant chemicals that induce extensive fungal cell death, primarily through regulated cell death (RCD). The surviving cells multiply within the plant dead tissue, indicating a shift from a localized to a spreading lesion. **B** Molecular response of susceptible and resistant plant cells to *B. cinerea* infection based on available information.

Infection caused by *B. cinerea* usually begins with oval conidia measuring 50–75 μm^3^ that adhere to and germinate on various plant parts, including leaves, flowers, and fruits [57, 58]. Germ tubes, which are elongated filaments, undergo specialization to transform into simple structures called appressoria and infection cushions (ICs). Both components serve to facilitate the invasion of the host. Additionally, it demonstrated that *B. cinerea* can invade the host by either entering through the stomata or by directly penetrating the cuticle using a short conidial germ tube [59]. Generally, *B. cinerea* completes the penetration by secreting toxic chemical, or cell wall degrading enzymes (CWDE). Two different phases have been discovered after the first host interaction: an early stage with isolated infection sites without dispersion, and a later stage with high fungal biomass and lesions (Fig. 1A). A new investigation into the dynamics of the disease has revealed the existence of an intermediate stage that was previously unknown, but is of paramount importance in the evolution of the disease [60]. The model projected by Shlezinger *et al.* [61] hypothesized that the initial stage of infection should cause the removal of a sufficient number of host cells, resulting in the development of dead tissue where fungal biomass can build up before advancing to the intermediate and late stages of infection. According to this hypothesis, the effective progression of the first stage of infection is highly dependent on the presence of chemicals that enhance the rapid destruction of host cells. In addition to the production of pathogenesis-related cell wall degrading enzymes (PCWDEs) and toxins, *B. cinerea* has the ability to produce several proteins known as cell death inducing proteins (CDIPs) [62], and, there is evidence that *B. cinerea* also uses the manipulation of the plant’s regulated cell death (RCD) machinery to effectively induce a localized host cell death [63]. However, as the infection moves from the immature to the middle stages, the fungus is subjected to substantial RCD triggered by antimicrobial chemicals found in plants [64]. At this stage, fungal life relies on the antiapoptotic machinery that prevents biomass destruction. The result of fungal development to the subsequent phase is contingent upon the equilibrium between the death of plant cells and fungal cells. Once the fungus successfully colonizes the host tissues and reaches a critical mass, the release of molecules that inhibit autophagy is replaced by the production of substances that inhibit the RCD [63]. This shift in molecular secretion ultimately leads to the destruction of plant tissue and facilitates the development of the disease.

**Figure 2:**
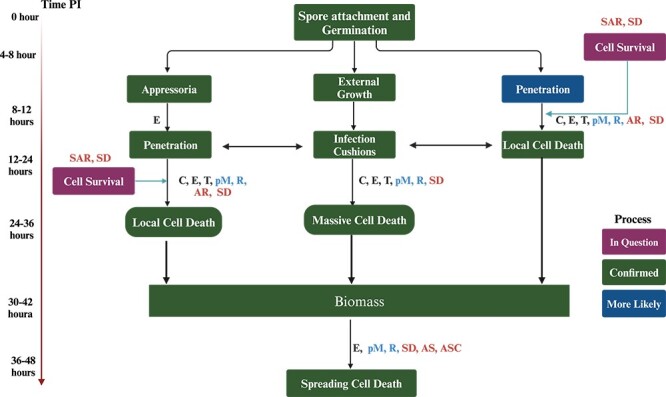
Roadmap of *Botrytis cinerea* infection. When conditions are ideal for the growth of a disease (such as an exposed host and tissue, enough water, and the right temperature), germ tubes can enter the outer layers either by themselves or with the help of appressoria. [Cell-death-inducing proteins (C), toxins (T), modulation of the pH (pM), enzymes (E) and the production of reactive oxygen species (R) suppress autophagy and/or regulated cell death (SAR), hypersensitive response (HR) and suppression of plant defense (SD)]. Whereas letters in black color represent where mechanism is confirmed, blue represents function is unclear and red color for predicted function.

The exact nature of the initial stages of disease establishment remains uncertain, as it is unclear whether there is a brief ‘biotrophic’ phase or an immediate induction of cell death [59]. Nevertheless, both models concur that these early activities lead to the establishment of an infection site that encourages the growth. Both ideas propose substances that trigger RCD aid lesion propagation (Figs 1 and 2). CDIPs and toxins have the capacity to cause both necrosis and RCD in the first phases of infection [65]. However, it should be noted that they do not play a role in the propagation of cell death. With the exception of oxalic acid, a compound known to induce RCD and perhaps facilitate the propagation of cell death, no additional molecules capable of causing RCD have been definitively found, although there is much evidence to support their existence. Before fungal growth, lesions expand with a perimeter of dead plant cells [61]. This sort of cell death is likely to be facilitated by fungal diseases, which may spread across the afflicted region (Figs 1A and 2). Furthermore, it is plausible that the dissemination of cell death may correspond to a process referred to as ‘runaway cell death’. This phenomenon is characterized by the unregulated propagation of cell death in plants following the initiation of the hypersensitive response (HR) [66]. In this scenario, the cell death process is initiated by CDIPs, toxins, or currently unidentified substances that activate the normally localized HR. The potential fungal toxins are responsible for the ongoing spread of cell death through their manipulation of HR regulatory systems [67]. Additionally, it has been shown that introducing anti-apoptotic genes into plants inhibits RCD and prevents infection [68]. All of these findings suggest that chemicals that elicit RCD promote the spread of lesions, and that necrotic cell death—possibly in conjunction with RCD—is the most likely cause of localized lesions.

Comparison between *B. cinerea* susceptible and resistant grapevine cells is shown in Fig. 1B. Research has shown that *B. cinerea* infection triggers the production of PCWDEs and CDIPs enzymes in infected cells. This occurs due to the down regulation of important genes connected with the JA/ET signaling pathway [69], whereas pH modulation fluctuates enzymes activity and increased grapevine susceptibility. In *Arabidopsis thaliana*, genes from *Vitis quinquangularis* and *Vitis amurensis* improved resistance to *B. cinerea* through the JA/ET signaling pathway and raised the transcript levels of genes associated to defense [70]. The majority of the *ERF* genes were up-regulated and suggested networks of genes that contribute to immunity in another study that examined the expression profiles of these genes at various times following *B. cinerea* inoculation [71] (Table 1). Grapevine infection with *B. cinerea* depends on JA/ET signaling, as well as on the build-up of stilbeneoids and the attenuation of hypersensitive cell death in cultivars that are susceptible as well as resistant [82] (detailed later Phytohormones). Similarly, the components that determine disease resistance are likewise the quantities of anthocyanin and phenolic chemicals. Greater disease resistance was demonstrated by the grapes with higher levels of catechin, epi-catechin, and delphinidin-3-glucoside [83]. Likewise, grapes infected with *Aspergillus niger* showed increased expression of the genes *STS* (Stilbene Synthase). Mass spectrometry analysis confirmed that the levels of pterostilbenes and trans-resveratrol were identified at minor levels, despite the higher gene expression [84].

**Table 1:** Grapes include genes that affect their response to *Botrytis cinerea*

Gene	ID	Chr	Start	End	Reference
*VvRbohD*	*VIT_201s0150g00450*	1	22 816 160	22 819 209	[72]
*VvGLP3*	*VIT_214s0128g00690*	14	3 215 490	0.216361	[73]
*VvPR10.1*	*VIT_205s0077g01530*	5	1 232 243	1 232 847	[74]
*VvPR10.2*	*VIT_205s0077g01580*	5	1 263 800	1 264 384	[74]
*VvPR10.3*	*VIT_205s0077g01550*	5	1 242 623	1 243 231	[74]
*VvERF066*	*VIT_14s0081g00730*	14	9 102 878	9 103 504	[71]
*VvERF099*	*VIT_18s0072g00260*	18	19 427 037	19 431 008	[71]
*VqTLP29*	*VIT_217s0000g02470*	17	2 254 359	2 255 331	[75]
*VaERF16*	*VIT_05s0077g01860*	5	1 463 118	1 464 392	[71]
*VvERF071*	*VIT_15s0021g01600*	15	12 230 432	12 231 049	[71]
*VvERF072*	*VIT_15s0021g01610*	15	12 235 112	12 235 735	[71]
*VvAP13*	*VIT_206s0061g01030*	6	18 652 672	1 865 856	[75]
*VqSTS9*	*Vv_216s0100g00770*	16	16 268 816	16 270 352	[76]
*VaSTS19*	*Vv_216s0100g00880*	16	16 368 410	6 366 907	[134]
*VqSTS21*	*Vv_216s0100g00910*	16	16 398 234	1 639 977	[77]
*VqSTS32*	*Vv16s0100g01040*	16	16 511 216	16 512 602	[76]
*VqSTS42*	*Vv16s0100g01140*	16	16 629 091	16 627 536	[76]
*WRKY3/VIWRKY3*	*VIT_201s0010g03930*	1	21 460 283	21 461 385	([74]; [78])
*WRKY57*	*VIT_219s0090g01720*	19	7 760 186	7 767 468	[78]
*WRKY58*	*VIT_219s0015g01870*	19	10 665 036	10 669 055	[78]
*VvSWEET2*	*–*	chr19.	278 703	280 954	[46]
*VvSWEET4*	*–*	chr14.	27 825 543	27 827 876	[46]
*VvSWEET7*	*–*	chr2.	1 857 989	1 860 568	[46]
*VvSWEET10*	*–*	chr17.	551 030	552 902	[46]
*VvSWEET11*	*–*	chr7.	2 367 917	2 369 607	[46]
*VvSWEET15*	*–*	chr1.	22 072 445	22 074 122	[46]
*VaPDF1.2*	*VIT_207s0130g00030*	7	20 130 371	20 131 439	[79]
*VvNPR1.1*	*VIT_211s0016g01990*	11	1 613 090	1 616 479	[80]
*VvXYLP02*	*VIT_205s0020g03740*	5	5 438 841	5 440 757	[81]

## Environmental determinants of *B. cinerea* infection

While the transmission of fungal infections under different conditions, such as temperature and relative humidity [85] remains poorly understood, temperature, relative humidity, precipitation and dew point have been noticed to play a vital role of *B. cinerea* infection in grapes [85].

### Relative humidity

Relative humidity (RH) is a critical factor in grape fungal infections because it directly affects the ability of pathogens to cause disease, germinate spores, form conidia, grow mycelium, and produce mycotoxins. Under high RH conditions, fungal spores reach a state of physiological maturity, end their dormancy, and promptly begin to germinate [86]. The process of germination varies depending on the location where it occurs, with an increased RH percentage triggering the germination and conidial development of harmful fungi affecting conidial production [87]. Increased RH may affect grape nutritional and bioactive component accumulation. As a result, spore germination process, conidial development and disease incidence in the prevalence of harmful fungi increase in correlation with RH [88]. Ciliberti *et al.* described that the growth/infection of *B. cinerea* was observed at 80–90% RH with temperature fluctuations [87]. Therefore, it is highly recommended to control RH during post-harvest placing and storage of grapes to minimize the occurrence of fungal infections.

### pH

Environmental variables, such as pH, have an impact on the disease harmfulness, development, and genomic regulation of numerous pathogenic fungi. *B. cinerea* secretes considerable quantities of organic acids, particularly oxalic acid, to acidify certain tissues during colonization. Moreover, it has the ability to raise the pH level of plant tissues during infection, most likely via releasing ammonia [89]. Because *B. cinerea* is an acidic fungus and the grape berries pH is 3.46 ± 0.20, it is unlikely that the acidity of the berries will affect the virulence of the fungus. The *BcpacC* deletion mutant of *B. cinerea,* on the other hand only differs in plants with neutral pH tissues. The results show that PacC affects OA synthesis and enzyme secretion, which affects the infectivity [90]. Apart from OA, aspartic acid proteases and polysaccharides in plant cells regulate their pH and influence *B. cinerea* pathogenesis in grapes [91]. These pathogenic factors play a role in altering host pH, promoting disease development, and producing mycotoxins.

### Water activity (a_w_)

Mycotoxins are influenced by water activity in grapes and grape products, which can lead to their proliferation, colonization, and accumulation. Water activity (a_w_) being less than 0.7 inhibits the proliferation of pathogens and microbial flora. However, raw grapes have a water activity greater than 0.95. Therefore, the presence of water in grapes promotes the proliferation of harmful fungi, and a_w_ needed for growth and mycotoxin production can vary [92]. The optimal a_w_ for growth and mycotoxin synthesis varies with the type of pathogen [93]. *B. cinerea* showed low to moderate growth under different a_w_ levels, while toxin production has not yet been clearly investigated [94, 95]. Further experiments are needed to fully understand the activity and development of *B. cinerea* under different a_w_ levels. In addition to water, temperature also has a significant effect on the growth and production of mycotoxins.

### Temperature

Temperature fluctuations can alter the occurrence and progress of fungal infections in grapes before and after harvest. *B. cinerea* did not exhibit any spread or symptoms at temperatures above 30°C. Nevertheless, at a temperature of 20°C, the fungus exhibited the highest disease occurrence [94, 96]. A model method shows that elevated temperatures accelerate the onset of disease outbreaks [87, 97]. These results suggest that variations in temperature between day and night, as well as the temperatures at which grapes are kept, might influence the growth of fungal diseases. To completely understand the impact of the grape microenvironment and forecast potential dangers associated with fungal infections, more research is necessary. [87].

## Defense of grapevine against *B. cinerea* infection

The life cycle of grapevine is modulated by several abiotic and biotic stresses. Currently, many grapevine genes have been found to resist or tolerate *B. cinerea* (Table 1). However, grapevine resistance or tolerance to *B. cinerea* is genetically unknown and requires more study.

### Physical barriers

**Figure 3:**
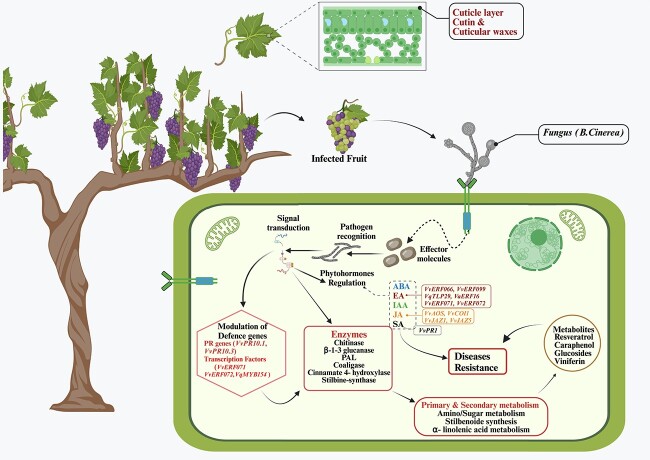
Resistance mechanism against fungal infection. The systematic elaboration of various pathways and genes involved in *Botrytis cinerea* resistance.

The plant cuticle serves as an initial defense mechanism against the germination of pathogenic conidia of *B. cinerea* (Fig. 3). The cuticle layer is mainly composed of two lipid types—cutin and cuticular waxes—which potentially interact with pathogens. It has been discovered that any restriction or alteration of these constituents in mutants resulted in variable gene activity during the initial phases of infection and consequently reduced the germination factor of *B. cinerea* [98]. The study conducted by Wan *et al.* [16] examined Chinese wild grape leaf *B. cinerea* resistance. The wild species with cell wall reinforcement have low infection rates and strong resistance to several fungal diseases such as gray mold, downy mildew, powdery mildew, and anthracnose. During the flowering stage, *B. cinerea* infects grapevine inflorescences by depositing airborne conidia that subsequently colonize the host cell surface [99]. *B. cinerea* infection of grapevine intensifies from green berry to maturity. Several gene families have been identified that regulate the biotic stress response in grapevine. During the early developmental phase of an unripe berry, the pathogen remains in the host tissues for an extended period of time without causing any visible symptoms [44, 100]. In the early stages of grape development, during the immature berry stage and the toward maturity stage, *GLPs* (germin-like proteins) were highly responsive genes in the presence of fungal infection, a grape *VvGLP* genes member *VvGLP3* activated at the cell wall after pathogen interaction [73]. The *VqDUF642* gene, derived from the Chinese grapevine accession Danfeng-2, plays a role in both berry growth and defense mechanisms against *Erysiphe necator* and *B. cinerea*. The resistance of berries in early development stages to *B. cinerea* has been attributed to various interrelated mechanical and chemical processes, although a comprehensive investigation of these processes is still lacking [16].

### Enzymes

Enzymes associated with the metabolism of phenolic compounds, phenylpropanes, lignin, and flavonoids may be responsible for the grape berries’ resistance mechanism against pathogenic fungi that invade at different growth stages [101, 102] (Fig. 3). Apple fruit responses to gray mold disease involve four enzymes: cinnamate 4-hydroxylase (C4H), phenylalanine ammonia-lyase (PAL), cinnamyl alcohol dehydrogenase (CAD), and 4-coumarate: coenzyme A ligase (4CL). The key processes in the phenylpropanoid pathway are catalyzed by the enzymes PAL, C4H, and 4CL, whereas CAD is important in the formation of the lignin precursor. Increased activity of these enzymes leads to an upregulation of lignin, flavonoids, and phenolic acid production. Furthermore, compared to Fuji fruits, which are prone to the gray mold disease, Qinguan fruits exhibit higher levels of enzyme activity [102]. The flavedo of citrus infected with *Penicillium digitatum* has greater expression levels of the genes encoding PAL, C4H, and CAD [101]. At the green berry stage, 15 PAL genes across the genome regulates *B. cinerea* infection in grapevine [103, 104], but further investigation is needed to uncover the genetic base of PAL in grapevine.

A naturally occurring phytoalexin with strong resistance to fungi-related diseases is resveratrol. A crucial enzyme in the manufacture of resveratrol is stilbene synthase (*STS*), which condenses one molecule of coumaroyl-CoA and three molecules of malonyl-CoA (coenzyme A) to generate resveratrol. Similarly, a genome-wide study in grapevine identified 48 *STS* (*VvSTS*) genes that mainly regulate *B. cinerea* infection [105]. These genes produce stilbenes (resveratrol and its derivatives) and lignin, which inhibit *B. cinerea* development, via the immunological pathway of phenylpropanoid biosynthesis [104, 105]. Two *STS* genes from wild grape named *VaSTS19* and *VqSTS21* antagonistically increase resistance to *B. cinerea*, while *VaSTS19* increase resistance against *B. cinerea,* whereas *VqSTS21* is involved in the susceptibility [77, 106]. Previously, *VaSTS19* from grapes was transferred to Arabidopsis, which increased the latter plant’s resistance to *B. cinerea* and powdery mildew [107]. Recently, Jian *et al.* [76] explained that the transcription factor *VqMYB154* from *V. quinquangularis* can activate the stilbene synthase genes *VqSTS9*, *VqSTS32*, and *VqSTS42* by binding directly to their promoters and to regulate the pathogen response in grapevine. The genes encoding putative lignin biosynthesis-related enzymes, such as cinnamoyl- CoA reductase (*CCR*) and p-coumarate 3-hydroxylase (C3H), were found to increase and enhance the levels of metabolites, including ferulic acid, affeic acid, and chlorogenic acid to combat fungal infection. A total of 69 *C3H* genes (named *VvC3H1- VvC3H6*) have been mapped in the genome of the grapevine [108]. The grapevine veraison berries exhibit cell wall reinforcement after *B. cinerea* infection due to the up-regulation of *VvC3H* genes. Their expression decreased at the ripe berry stage. Kelloniemi *et al.* [109] found that the overexpression of *CCR* genes was associated with cell wall reinforcement and the production of the antimicrobial compound resveratrol in unripe grape berries to counter *B. cinerea* infection. Similarly, strawberry (*Fragaria vesca*) leaves infected with *B. cinerea* showed increased expression of the majority of *CCR* genes [109, 110]. The *VvEXT* gene, responsible for encoding a protein rich in proline and similar to extension, exhibited an increase in expression levels throughout the green berry stage.

The phospholipase D (*PLD*) family controls phytohormone signaling, PCD, root morphology, and environmental stress responses in plants [111]. The structural study revealed that the *PLD* gene family can be divided into six distinct subgroups: *α*, *β/γ*, *δ*, *ε*, *ζ*, and *φ*, which are descended from four ancestors due to gene sequence duplications [112]. According to reports, *OsPLDβ1* from *Oryza sativa* is important in defense response and disease resistance, but *OsPLDβ2* is essential for regulating the ROS-scavenging enzyme at high temperatures [113, 114]. In Arabidopsis the defense signaling of powdery mildew fungus was mediated by *PLD* genes, and *PLDδ* was the primary isoform implicated in penetration resistance against pathogen [115]. In grape, 11 genes encoding *PLD* were identified. The *VvPLD* genes were further analysed and categorized into six different categories *(VvPLDα*, *VvPLD β/γ*, *VvPLDδ*, *VvPLDε*, *VvPLDζ*, *VvPLDφ*) and three groups based on sequence variation (*C2-PLD*, *PXPH-PLD*, and *SP-PLD*). The *VvPLD* genes named *VvPLDβ1*, *VvPLDβ2*, *VvPLDδ2*, *VvPLDρ*, and *VvPLDζ* were up regulated at 6 to 9 hours after *B. cinerea* infection, whereas the expression of *VvPLDa* and *VvPLDd* was suppressed [116]. The identified genes can be considered as potential candidates for resistance genes and can be used to develop grapevine lines that are resistant to *B. cinerea*.

The grape genome has a total of 50 aspartic protease (AP) genes, collectively referred to as *VvAP.* The *VvAP13* mutant exhibited susceptibility to *B. cinerea* [75]. Dadáková *et al.* [74] examined five different transcripts of a pathogen-related protein gene named *VvPR10*; the gene transcripts that showed the highest level of expression were *VvPR10.1*, *VvPR10.3*, and *VvPR10.9* at 24 hpi (hour post inoculation) and they play a pivotal role in resistance to *B. cinerea*. In grapevine, the *VvWRKY33* genes influence the expression of *VvPR10.1*, a grapevine gene regulates resistance to *Plasmopara viticola*.

Plants contain a diverse family of Thaumatin-like proteins (*TLP*), each with distinct functions and variable responses to abiotic and biotic stresses [117, 118]. A broad range of resistance to several diseases, such as *Elsinoe ampelina*, *Verticillium dahliae*, and certain filamentous fungus, such *B. cinerea* and *Fusarium oxysporum*, is attributed to certain *TLP* genes [119, 120]. Tobacco overexpressed a novel gene called *PpTLP* that was identified from the fruit of the ‘Huobali’ pear, and the transgenic plants suppressed the growth of different fungal pathogens in their hyphae to differing degrees [121]. A total of 33 *TLP* genes (*VvTLP*) were analysed in *V. vinifera* L. to evaluate their disease resistance. Overexpression of *VqTLP29*, a gene derived from the ‘Shang-24’ cultivar, in *A. thaliana* resulted susceptible to *B. cinerea* [122]. Similarly, it has been observed that overexpressing *Camellia sinensis* TLP, or CsTLP, in potatoes increases their resistance to fungal infections [123]. Through a variety of regulatory strategies, *TLPs* may strengthen a plant’s defenses against diseases like *B. cinerea*. Additional research is needed to determine how other member of *TLPs* genes regulate diseases in grapes.

One of the biggest families of plant sugar transporters, Early-Response to Dehydration six-like (*ERD6l*), is also one of the least investigated, with relatively few members that have been fully characterized. The *ERD6l* family, which all localize in the tonoplast, was functionally identified in Arabidopsis as monosaccharide transporters. Additionally, their expression changed in response to several abiotic stressors such as heat, salinity, ABA therapy, wounding, and zinc stress [124, 125]. Recently, Breia *et al.* [126] identified 18 members of the grapevine *ERD6l* family, functionally characterized *VvERD6l13*, and reported that its expression was up-regulated after berry contact with *B. cinerea*. *VvERD6l13* is expressed in leaves, stems, and flowers significantly in roots. The H + -dependent transport of sucrose (Km = 33 mM) is mediated by *VvERD6l13*, which is localized at the plasma membrane. As a result, the position of *VvERD6l13* as a valid sugar transporter that is involved in sugar mobilization in grapevines and is transcriptionally activated in response to biotic stress has been strengthened.

The recently revealed high-capacity, low-affinity sugar transporters known as *SWEETs* play vital roles in a variety of physiological processes where sugar efflux is essential. Pathogens find *SWEETs* to be attractive candidates for manipulation, and during an infection, their expression may undergo transcriptional reprogramming. In *V. vinifera* 17 genes encodes *SWEET* protein were identified in a genome-wide study. On the other hand, the presence of the necrotrophic pathogen *B. cinerea* resulted in a considerably higher level of expression of *VvSWEET4*. The *Arabidopsis* gene called *AtSWEET4* was discovered to be vulnerable to *B. cinerea* in knockout mutants [46]. *VvSWEET* mobilizes sugars throughout grape berry development and is transcriptionally altered by *B. cinerea* infection. In the *Botrytis*-susceptible cultivar ‘Trincadeira,’ *VvSWEET7* and *VvSWEET15* are considered to have an important role in fruit development and *Botrytis* infection after inoculation with fungal pathogen*.* This is because they show significant expression during the immature berries and mature berries stages, respectively and were apparently increased in response to infection. Moreover, the infection caused by *B. cinerea* resulted in a down-regulation of *VvSWEET17a* during the green stage, *VvSWEET10* and *VvSWEET17* during the veraison stage, and *VvSWEET11* during the mature stage [22].

Plant growth, development and stress responses in plants need the presence of genes encoding protein xylogen-like proteins (XYLPs). Nevertheless, there is little knowledge about the grape XYLP gene family and its role in providing protection against gray mold. In *V. vinifera* the genome has six XYLP genes, which were named *VvXYLP01*–*VvXYLP06* based on their location on the chromosomes. It was found that the expression level of *VvXYLP02* in resistant genotype greatly increases during *B. cinerea* infection, while the expression level in the sensitive genotype showed a segregating pattern. An *A. thaliana* overexpression line with *VvXYLP02* showed an upregulated response to *B. cinerea* [81].

Chitosan may significantly affect *B. cinerea*-resistant plants. Chitosan prevents infection via the JA (jasmonic acid) signaling pathway, activating defense genes and preserving berry quality. By inhibiting the expression of the negative regulators *VvHDAC19* and *VvTPR3*, both collectively play role in the production of fruit disease resistance; the application of chitosan led to a varied resistance to *B. cinerea*. JA and chitosan block *VvTPR3-VvHDAC19* interaction, suppressing their activity. The synthesis of phenolic chemicals resulted in the reinforcement of the cell wall. JA production in ripe fruits was increased and oxidative stress was controlled. Moreover, the proliferation of *B. cinerea* was suppressed [127]. In addition to enhancing the disease resistance of plants, these plentiful gene resources can also boost the development of plants and the quality of their fruits. Moreover, the disease known as gray mold can be controlled on grapevine crops by employing certain amounts of natural substances.

### Phytohormones

Plants use various internal mechanisms, such as the production of phytohormones like cytokinins, salicylic acid (SA), abscisic acid, jasmonic acid (JA), gibberellic acid (GA), ethylene (ET), brassinosteroids (BA), indole-3-acetic acid (IAA), and strigolactones (SL), to withstand challenging conditions (Fig. 3). Phytohormones regulate plant growth and development in response to stresses both biotic and abiotic. They act as a crucial internal signaling agent that mediates numerous molecular, biochemical, and physiological responses through a stress-responsive regulatory cascade. In response to infection, various genes expressed differently, mainly those involved in the production and communication of phytohormones [128]. Following the plant contact with fungal elicitors, their synthesis (hormone-regulating genes) in plant tissues begins quite rapidly [129]. Systemic acquired resistance (SAR) is linked to the development of local resistance to biotrophic infections (Figs 1 and 2). Biotrophic pathogens typically elicit SA-mediated defense response, which triggers a number of subsequent physiological immunological responses, including PCD and ROS accumulation [130]. ET and JA regulate the susceptibility to necrotrophic pathogens and induce systemic resistance. Infections that are semi-biotrophic cause signaling reactions mediated by both SA and JA [128]. Grape and *B. cinerea* interactions were significantly affected by phytohormones ET, JA, and SA [79]. Moreover, the role of GA and ABA in controlling *B. cinerea* in grapes has been recognized [70]. There is a suggestion that the routes facilitated by SA and JA exhibit antagonistic behaviour (Fig. 1). When plants initially activate the SA-dependent system to protect against a specific pathogen, this results in inhibition of JA signaling, i.e., *VaRGA1* in *V. amurensis* ‘Shuanghong’ induced by SA, but inhibition by JA results in enhanced resistance to *B. cinerea* [131].

The defensive response against a variety of diseases and pests often involves the JA pathway. In ripe berries*, VvJAZ1*, which is involved in the enzymes of the JA biosynthesis pathway, is highly activated [132]. Internal JA triggered a defense response in grapevine species that are vulnerable to *B. cinerea* [133], and regulate *VvPR1* gene expression; the expression of *VvPR1* was increased under SA treatment, indicating that SA may be modulated in basal resistance maturation (Figs 1 and 3). In Chinese wild grape *V. quinquangularis*, three ERF-associated genes—*VqERF112*, *VqERF114*, and *VqERF072*—showed heterogeneous responses to phytohormones treatment [70]. A *WRKY57* mutant modulates resistance to *B. cinerea* by interacting with *VvCOI1* in the JA signaling pathway. *WRKY33* interacts directly with *VvJAZ1* and *VvJAZ5* but another *WRKY* gene, *WRKY57*, binds to the promoter of *VvJAZ1* and *VvJAZ5*. *WRKY57* and *WRKY33* exert competing regulation over *VvJAZ1* and *VvJAZ5*. *WRKY57* undermines the resistance of *B. cinerea* by competition with *WRKY33* to regulate the transcription of *JAZ1* and *JAZ5*. Eulgem *et al.* [78] reported that *WRKY33* functions as an inhibitor of the transcription of *VvJAZ1* and *VvJAZ5*. *A. thaliana* plants that expressed genetically modified *VlWRKY3* genes exhibited heightened susceptibility to *B. cinerea*. This might be attributed to the interplay between the MeJA and SA signaling pathways, or the impact of other genes. Prior studies have demonstrated that WRKY3 reacts to MeJA and ET, as well as to salt and drought stress [134].

The *VvAOS* and *VvCOI1* genes have significant roles in the biosynthesis and signaling of JA. These genes influence berry ripening by regulating fruit color. They also have an impact on berries’ vulnerability to *B. cinerea* infection [127]. JA accumulates rapidly in plant organs following injury or interaction with fungal pathogens. Knockdown or any variation in the *VvAOS* gene results in a reduction in the JA content [135]. Similarly, the samples exhibiting increased expression of the *VvCOI1* gene (*VvCOI1-OE*) were distinguished by a substantial elevation in JA levels and a noticeable postponement in the fungal infection. In strawberry, increased expression of the *COI1* gene had no direct effect on the JA levels, but stimulated the activation of plant important enzymes that play a critical part in protecting fruit against gray mold infection [127]. Four *CRK* genes have been identified in wild grape *V. amurensis*. Increased expression of *VaCRK2* in *A. thaliana* increases resistance to *B. cinerea*. After *B. cinerea* inoculation and MeJA treatment, a gray mold-resistant grape cultivar exhibited considerable *VaCRK2* gene upregulation*.* The genes that are activated by *VaCRK2* and are related with the JA signal initiation pathway, genes implicated in PR, and the buildup of ROS are expressed [136]. The promotor TGA element and the AuxRR core have been linked to the auxin response. The response to ABA involves the ABRE element, the response to SA involves the TCA element, and the response to ET involves the ERE elements. *VvERF099* has a P-box motif that is sensitive to gibberellin [137]. However, *plant respiratory burst oxidase homologues* (*Rboh*) genes appear to be important for hormone signaling, defense, and development. In grapevine, seven putative *VvRboh* genes have been identified and are involved in regulating stress response [72], and the gene *VvrbohD* shows up-regulation under *B. cinerea* and powdery mildew infection [138].

Abscisic acid (ABA) functions in opposition to *B. cinerea*, but cytokinin (CTK) contributes positively to resistance against it. There have also been reports of the other hormones in crops. Genes linked to the ‘ABA biosynthesis process, ABA catabolic process’, and ‘response to ABA’ were significantly increased when *B. cinerea* infected *V. vinifera* [139]. In order to combat *B. cinerea*, AUX, CTK, GAs, ABA, ET, brassinosteroid, JA, and SA signal transduction have altered in grape berries and kiwifruits [33]. These highlights demonstrated the crucial moments for implementing hormones in present-day agriculture to fully exploit their potential in enhancing crops and changing modern agricultural practices. Further investigation is needed to uncover the complete role of these hormones.

### MAMPs

In plants, protective immunological signaling is activated by pathogen-derived microbial-associated molecular patterns (MAMPs) and host damage-associated molecular patterns (DAMPs). When a plant is abiotically stressed or infected with a pathogen, its tissues emit endogenous messenger proteins, or DAMPs [140, 141]. So far, most of the knowledge about MAMPs and DAMPs comes from the model organism Arabidopsis, while only limited research has been conducted with grapevine. Following a *B. cinerea* infection, the plant cell wall (CW) releases oligogalacturonides (OGs), which act as DAMPs and support the plant’s defense response against the pathogen [142]. The overexpression of receptor wall associated kinase 1 (*WAK1*) enhances Arabidopsis resistance to *B. cinerea* via detecting DAMPs [143]. Endopoly-galacturonases (endo-PGs) activity is restricted by plant polygalacturonase-inhibiting proteins (*PGIPs*). Transgenic plants that overexpressed *PGIP*-encoding genes demonstrated increased pectin breakdown, which lessened disease symptoms associated with *B. cinerea*. Plants carrying the Arabidopsis receptor-like protein 30 (*rlp30*) mutation exhibited heightened vulnerability to both *B. cinerea* and *Sclerotinia sclerotiorum*. *RLP30*-mediated signaling requires the two *RLPs*: *BAK1* (BR insensitive1-associated receptor kinase1) and SOBIR1/EVR (suppressor of *BIR1*/evershed) [144]. This suggests that *B. cinerea* can infect mutants lacking *BAK1* and *SOBIR1*. Botrytis-induced kinase1 (*BIK1*) in Arabidopsis encodes a cytoplasmic kinase (*RLCK*) that functions as a receptor and promotes pathogen-derived MAMP-triggered immunity (*PTI*) in the early stages of *B. cinerea* infection [145]. Arabidopsis was more vulnerable to necrotrophic fungi when the *BIK1* gene was mutated [146].

**Figure 4:**
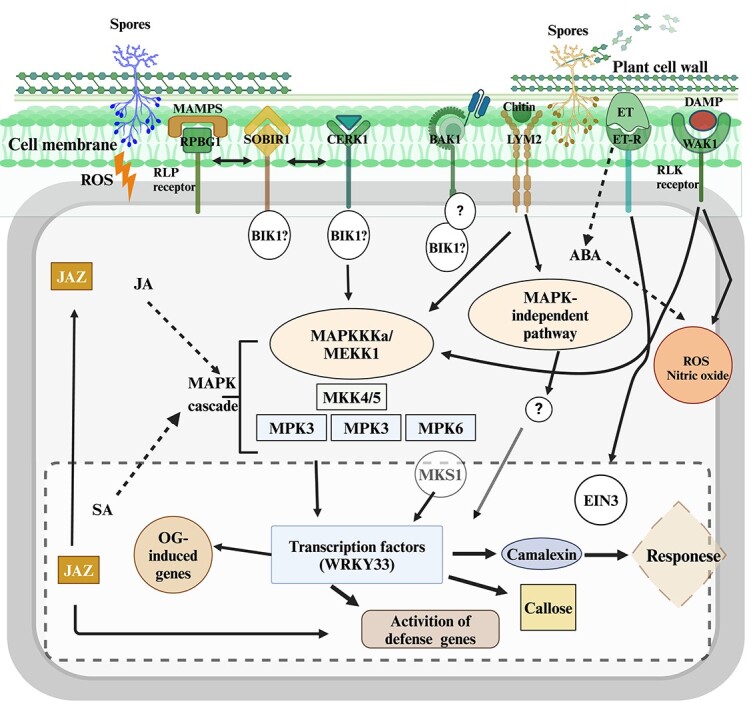
Diagrammatic representation of plant defensive mechanism against *Botrytis cinerea* infection.

After receiving signals from PRRs, MAPK kinase kinase (MAPKKK) activates MAP kinase kinase (MAPKK). Target proteins are then activated as a result of the activated MAPKK activating MAP kinase (MPK). *SCFE-1* activates MPK3, MPK4, and MPK6, indicating their significant roles in plant immunity (Fig. 4). For example, it was observed that Arabidopsis mpk3 mutants showed increased vulnerability to *B. cinerea* [147]. Additionally, it was demonstrated that *AtMPK6* is necessary for the elicitor-induced resistance against *B. cinerea* [148]. During a *B. cinerea* infection, the formation of camalexin and the biosynthesis of ET need Arabidopsis *MPK3* and *MPK6*. Moreover, *MPK3* and *MPK6* control the activities of CYP81F2 and the IGS O-methyltransferases*, IGMT1* and *IGMT2*, via the MPK3/MPK6 substrate ethylene response factor 6 (*ERF6*). When plants are infected with *B. cinerea*, the MAPS substrate 1 (*MKS1*), a substrate of MPK4, negatively controls plant defense. The mkp2 mutant of MAPK phosphatase 2 (*MKP2*) exhibits heightened vulnerability to *B. cinerea* [149]. While MKP2 can interact functionally with both *MPK3* and *MPK6,* its roles in controlling these two kinases differ when exposed to fungal elicitors. After infection by *B. cinerea*, MAPKs are phosphorylated many times, resulting in alterations to transcription, including TF-encoding genes [150]. MAPKK genes of grapevine have been over-expressed in Arabidopsis to assess their impact on plant ability to withstand abiotic stress. The results demonstrated that overexpression of VvMKK2 and VvMKK4 genes resulted in an improved tolerance to salt stress, a higher germination of seeds, higher survival rate, and better seedling growth under stress conditions than wild-type plants. In addition, overexpression of VvMKK2 enhanced drought stress tolerance [151]. However, further investigation is required to determine the specific genes in the MAPKs grapevine pathway that respond to *B. cinerea*. For instance, *WRKY33* positively controls the gene *PAD3*, which is involved in the biosynthesis of camalexin, and is necessary for Arabidopsis defense against *B. cinerea* [152]. According to reports, when a pathogen is encountered, MPK4 is activated, which phosphorylates *MKS1*. Phosphorylated *MKS1* then causes MPK4 to release *WRKY33*, which in turn causes PAD3 to become more active [153].

### Transcription factor

In plants, the ethylene-responsive factor (*ERF*) gene family is essential for plant growth and defense networks [154–156]. Zhu *et al.* [71] identified 113 *ERF* genes in *V. vinifera* (*VvERF*), and 35 ERF genes showed segregation in response to *B. cinerea*, indicating their role in plant defense [71]. Another member of *VvERF, VaERF16* from the Chinese wild grape (*V. amurensis* ‘Shuang You’), is expressed during *B. cinerea* infection. *VaERF16* interacts with *VaMYB306,* which increases the transcript levels of *VaPDF1.2,* ultimately enhancing the response grape leaves to *B. cinerea* infection [79]*.* The *cis*-elements in the promoter revealed that most *ERF* genes possess CGTCA motif and TGACG motif elements. These elements are known to be involved in the response to MeJA, especially in the case of *VvERF071* and *VvERF072*. These results imply that these genes may have a role in the immune system’s reaction to fungus-related infections.

NAC family transcription factors have a role in diverse developmental processes and respond to environmental stressors. Arabidopsis plants demonstrate increased resistance to osmotic, salt, and cold stressors, as well as to *B. cinerea* and *Hyaloperonospora arabidopsidis* infections. These plants have an altered pattern of defense gene markers (*AtPR-1*, *AtPDF1.2*, and *AtVSP1*) following the application of stress, indicating that *VvNAC1* plays a crucial role in regulating the plant’s defense signaling pathway [157]. Arabidopsis *ATAF1* functions as a suppressor of defensive mechanisms against both necrotrophic fungal and bacterial infections [158]. *ATAF1* expression was reduced following infection with *B. cinerea* or *Pseudomonas syringae pv*. tomato, as well as after exposure to plant hormones. Conversely, transgenic plants with overexpressed *ATAF1* gene (*ATAF1-OE*) displayed heightened vulnerability. On the other hand, the expression of an *ATAF1* chimeric repressor construct (ATAF1-SRDX) resulted in increased resistance against *P. syringae* pv. tomato DC3000, *B. cinerea*, and *Alternaria brassicicola* [159]. Recent investigation has uncovered a significant finding: 10 distinct *VvNAC* genes have been associated with resistance to *B. cinerea* [160]. Furthermore, when the *SlSRN1* gene was silenced in tomato plants, they became more vulnerable to *B. cinerea* infection [161]. Similarly, when the *StNTP1* and *StNTP2* genes, which are part of the NTL subfamily of membrane-localized NAC transcription factors, were silenced in *Nicotiana benthamiana*, they became more susceptible to *Phytophthora infestans* infection [162]. The molecular basis of the actions of NAC transcription factors in immunity and their potential application has role in enhancing crop disease resistance.

The introduction of *VaERF16* from grapes into *A. thaliana* significantly increased its ability to resist *B. cinerea* and the bacteria *P. syringae DC3000*. This enhancement was achieved through the activation of the salicylic acid and jasmonate/ethylene signaling pathways. Understanding this regulatory module could be valuable in improving grapevine resistance to *B. cinerea* infection [79]. Another transcription factor, *MYB46*, plays a crucial role in determining the susceptibility of plants to the fungal pathogen *B. cinerea*. The researcher discovered *MYB46*’s capability to attach to a novel *cis*-element situated in the 5′ promoter region of the *Ep5C* gene, which is activated by pathogens. This gene encodes a type III peroxidase that is bound to the cell wall. The genetic and molecular evidence suggests that *MYB46* regulates the extent of *Ep5C* gene activation in response to pathogenic attacks [163]. According to Gao *et al.* [164], *BjMYB1* has been identified as a binding agent for the Wbl-4 domain located in the promoter region of the *BjCHI1* chitinase gene. By overexpressing *BjMYB1*, hosts gain resistance against the fungus *B. cinerea* through the activation of *BjCHI1*.

WRKY transcription factors also have significant functions in plant immune responses. Nevertheless, there is currently a scarcity of information regarding the *WRKY* gene family in plants. Empirical research documented a total of 23 *LrWRKY* genes containing fully intact *WRKY* domains in the Botrytis-resistant plant species *Lilium regale*. Overexpression of *LrWRKY4* and *LrWRKY12* genes led to the development of plants that exhibited enhanced resistance against *B. cinerea* compared to the normal wild-type plants [165]. *B. cinerea* increased tomato SlDRW1 expression by 10–13 times compared to mock-inoculated plants, whereas *P. syringae pv.* tomato (Pst) DC3000 did not. Silencing SlDRW1 increased *B. cinerea* disease severity but did not impact Pst DC3000 disease phenotype. Similarly, the grapevine gene *qWRKY52*, responsible for encoding a member of the *WRKY III* gene family, was expressed in Arabidopsis, it improved resistance against powdery mildew but made the plants more susceptible to *B. cinerea*, in comparison to the wild-type plants [134]. Another grapevine *WRKY* family gene, *VlWRKY48*, governs several reactions to drought stressors and boosts immunity against powdery mildew infection [166]. Wang reported the overexpression of *VvWRKY18* of grapevine increased the expression of *STS* and *PR* genes, so enhancing the defense response of transgenic plants and thereby suppressing the invasion of *B. cinerea* [167].

### Epigenetic regulation

Epigenetics pertains to the dynamic alterations in the chemical makeup of the genome that can sustain gene expression without any mutations to the DNA sequence. Epigenetic pathways are crucial in the processes of growth and development [168]. Existing research indicates that decreased DNA methylation enhances the reactivity of the plant’s immune system. There is evidence suggesting that DNA methylation has a function in grapevine’s interaction with the necrotrophic pathogen *B. cinerea*. Analysis of gene expression in grapevine berries showed that genes responsible for epigenetic alterations, including DNA (cytosine-5)-methyltransferase, helicases, DICER, and ARGONAUTE proteins, exhibited differential expression in response to *Botrytis* infection [169]. Nerva *et al.* showed that the application of double-stranded RNA (dsRNA) using spray-induced gene silencing (SIGS) can effectively enhance plant resistance against *B. cinerea* in the ‘Moscato’ cultivar, which is grafted onto Kober 5BB rootstock [170]. The involvement of double-stranded RNAs (dsRNAs) in the process of RNA-directed DNA methylation has been extensively shown. In Arabidopsis, DNA methylation inhibited crown gall tumors induced by the soilborne biotrophic pathogen *Agrobacterium tumefaciens*, which causes crown gall disease in grapevines [171]. The assessment of the connection between the DNA methylome and transcriptome revealed 144 genes that exhibited a negative correlation between promoter methylation and gene expression. These genes were mostly associated with responses to biotic stress and the production of flavonoids in grapes barriers. In addition, the use of 5-azacytidine and melatonin resulted in comparable impacts on the growth of *B. cinerea* mycelium, the rate of decay in berries, and the production of flavonoids. In addition, *EDS1* was employed to demonstrate that melatonin enhanced gene expression by reducing levels of promoter methylation [172]. Xu *et al.* reported sodium valproate demonstrated effectiveness against *B. cinerea* by promoting the acetylation of histone H3, specifically H3K9ac, H3K14ac, and H3K56ac in tomato [173]. Another study found a combined sum of 1 169 852 and 1 008 894 methylated cytosines (mCs) were detected in the control and melatonin-treated grape berries, respectively. The majority of mCs were found at CG sites, followed by CHG sites and CHH sites. In comparison to the control group, the administration of melatonin resulted in a significant reduction in methylation levels at CHG and CHH sites in different gene areas in grape wine. Similarly, the levels of H3K9ac mark have significantly increased in the early induced genes SlyDES, SlyDOX1, and SlyLoxD, which encode enzymes involved in the oxylipin pathway, as well as in the gene SlyWRKY75, which codes for a transcriptional regulator involved in hormone signaling. This rise in H3K9ac mark is observed in the tomato’s response to *B. cinerea* [174].

Epigenetic mechanisms are triggered in plants not only during harmful infection, but also during the symbiotic association of plants with endophytic fungi, with the purpose of preserving advantageous interactions and fostering symbiosis. Similar to MAMP research, the main body of knowledge on epigenetic regulation also comes from other model plants such as Arabidopsis. In the future, more emphasis could be ‘grafted’ to grapevine research for the development of disease resistance in grapevines.

## Disease management and development of resistant species with new gene-editing technology

Investigations into the relationship between fungal infections and grape defense help viticulture create effective disease control measures. Traditionally, grapevines have been protected against fungal infections by the use of chemical fungicides, biological control agents, and physical treatments [175]. The efficacy of preharvest spraying as a method for disease prevention in grapes has been well studied. Prior to harvesting, the fruit’s surface is treated with functional coatings to enhance fruit quality and prevent disease. An antifungal activity test was conducted on a nanocomposite material comprising of poly (ethylene-co-vinyl acetate), chitosan, and iprodione. The chitosan application improved on fruit quality [176]. The nanocomposite material has substantial benefits, including reducing pesticide use and improving grape quality. [177] research suggests that a non-toxic food additive—ε-PL—can effectively prevent grape gray mold degradation in grapes. To prevent gray mold and safeguard grapes from diseases, ε-PL produced membrane damage in the pathogenic fungus.

Plant essential oils (PEOs) are another option for preventing grape diseases. PEOs are beneficial disease control agents due to their antioxidant properties, safety, broad-spectrum antibacterial and antifungal capabilities, and the difficulty for microorganisms to build resistance. *Melaleuca alternifolia*, *Origanum dictamnus*, *Origanum onites*, *Thymbra capitata,* and *Satureja thymbra* oil extract showed active control of fungal infections [175, 178].

Recent research has demonstrated the inhibitory effects of a number of bacterial strains against pathogenic fungi. Microbe origin biocontrol products may compete with one another for nutrients and habitat, and they may also produce volatile organic compounds (VOCs), which can inhibit the growth of pathogens and strengthen human defenses against disease [179]. A study conducted in 2020 [180] showed that some yeasts, including *Cryptococcus magnus*, *Aureobasidium pullulans*, *Rhodotorula glutinis*, and *Metschnikowia pulcherrima*, obtained from grape must and the surface of grapes had remarkable efficacy in controlling *Penicillium expansum* disease in grapes. Several yeast species, including *Pichia*, *Saccharomyces, Metschnikowia*, *Rhodotorula*, and *Dekkera* were found to effectively inhibit the development and production of OTA (ochratoxin A) by the grape fungus *Aspergillus carbonarius* via competition [181]. The present findings showcase the capacity to manage numerous grape diseases produced by pathogenic fungi and diminish the occurrence of mycotoxins in grapes and associated products, and at the same time preserving their quality.

**Figure 5:**
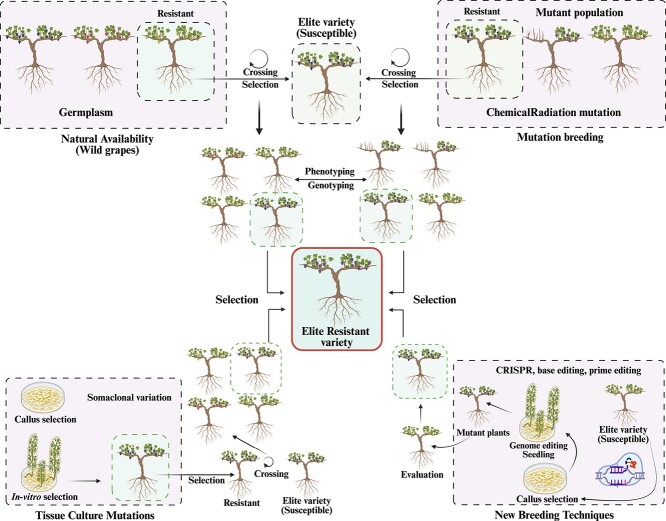
Progression of agricultural breeding methods. The process of crossbreeding takes a significant amount of time, often 8–10 years, to improve desirable characteristics or features in a particular species, such as disease tolerance or resistance. Mutation breeding uses chemical or physical irradiation to develop unique genetic variants in the genome over 6–7 years. Tissue culture improves crop attributes in 4–6 years by exogenously transforming genes into commercially relevant elite cultivars. Genome editing: precisely updating the target gene or regulatory sequence or modifying elite kinds’ DNA and/or RNA bases in 2–3 years to improve a specified characteristic.

Human selection has resulted in a wide range of cultivars with beneficial traits related to crop productivity, timing of growth stages and chemical composition of the berries. However, these rigorous breeding methods have led to the depletion of several other traits, such as the ability to withstand biological stresses, in cultivated genetic resources that were originally found in wild relatives of crops [182]. As a result of the massive attempts to generate resistant varieties, researchers are looking for gene and genome-based breeding methods. An effective strategy involves the integration of classical selection and MAS to introduce specific genetic loci into novel types [45]. The MAS accelerate up and lower the cost of selection. Additionally, it guarantees the development of varieties with novel gene combinations, a task that is almost unattainable with conventional breeding techniques but still requires a significant amount of time to achieve the desired results [183]. Next, tissue culture techniques are required to regenerate seedlings from transformed cells. This process is laborious and often takes several months. It often involves the use of costly and environmentally harmful substances such as antibiotics and herbicides in the growth medium to identify the potential transgenic plants [184]. New breeding technologies (NBTs) have become increasingly prominent in recent years. These technologies, including gene editing (GE) using zinc finger nucleases (ZFNs), transcription activator-like effectors nucleases (TALENs) and CRISPR-Cas-associated nucleases offer innovative ways to improve the genetic traits of important agricultural crops [47]. Compared to conventional methods, NBTs are efficient and require less time to achieve the desired results (Fig. 5).

Advances in plant genetic engineering and genomic research offer a promising avenue for creating novel plant varieties that are well suited to meet the demands and problems of the modern market under adverse weather conditions [185]. In this regard, grapevine improvement is mainly achieved through the use of both traditional and precision breeding techniques. Recently, research has been actively involved in the development of genetic engineering methods [186]. The grapevine genome has been sequenced, revealing the presence of new genes, structural gene variations, single nucleotide polymorphisms, and regulatory elements. These findings facilitate the development of resistant grapevine lines through precision breeding using genetic engineering techniques [187]. These days, the CRISPR/Cas9 system—also known as the clustered regularly interspaced short palindromic repeats/CRISPR-associated protein 9 system—is one of the most successful genetic engineering techniques. This technique allows the precise creation of specific changes in the genome by direct manipulation of DNA. The Cas9-induced double-strand break in the DNA activates DNA repair pathways such as homology-directed recombination and non-homologous end joining. Guide RNAs (gRNAs) help Cas9 identify target-specific sequences more effectively [187, 188]. To edit a genomic target-based synthetic RNA fragment of roughly 20 nucleotides, a scaffold sequence is needed for Cas binding [188]. If the desired reign is close to a protospacer adjacent motif (PAM) that is three base pairs long, Cas9 will eventually generate double-strand breaks (DSBs). This allows the proposed DNA repair pathways to function, resulting in target-specific mutation or editing [188].

**Figure 6:**
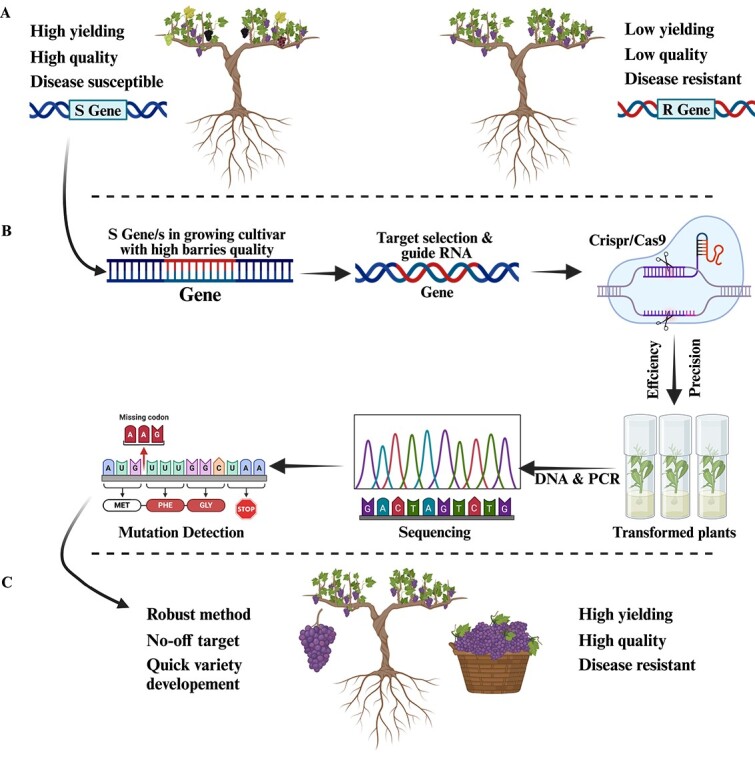
A systematic approach for CRISPR application in grapes for disease resistance by targeting susceptibility (S) gene(s). **A** Identifying a cultivar with favorable features except disease resistance and functioning S gene(s) for the disease(s). **B** GETs like CRISPR/Cas9 may disrupt S gene(s). It accurately cuts a guide RNA-guided site. Plants with the required phenotype may be created more quickly and effectively utilizing GETs. **C** Ultimate disease-resistant cultivar. CRISPR/Cas9 is more reliable, efficient, and time-saving for S gene disruption and disease-resistant variety generation.

CRISPR/Cas9 gene editing has been documented in grapes since 2016 (Fig. 6). The process involves the use of binary Ti-derived plasmids that are compatible with Agrobacterium-mediated gene transfer. These plasmids are used to express gRNA and Cas9 mRNA. Wang *et al.* [37] first utilized CRISPR GE to construct mutant variants of the *VvWRKY52* transcription factor gene in both mono- and bi-allelic states. This resulted in the production of regenerated plants with increased resistance to *B. cinerea*. More recently, Li *et al.* [189] generated a loss of function mutant of the *VvPR4b* gene in the Thompson Seedless grape cultivar; the mutant showed increased susceptibility to the pathogen. While some herbaceous species can benefit from *in vitro* plant regeneration of protoplasts, several variables hinder the development of this technology in resistant woody fruit plant species. To date, *VvWRKY52* edited via Crispr found increased resistance in grapevine. Efforts in grapevine are relatively recent and require further research.

## Implications for viticulture and future recommendations

Global food and nutrition security is threatened by climate changes such as temperature extremes, flooding, and soil erosion. These adverse climate changes are likely to reduce grapevine growth and production. This review highlights the need to advance omics technologies in cultivated grapevines to improve grapevine genetics. This advancement will allow us to implement targeted tactics that effectively maintain and sustain vineyard productivity. The identification molecular genetic markers and genetic networks linked to disease resistance may help identify genotypes with improved disease tolerance. Creating an inhospitable environment for *B. cinerea* will limit its growth and reduce the incidence of infection both in the field and in storage. Whole-genome sequencing of infectious fungi combined with bioinformatics can speed up the identification of genetic variants that specifically target certain illnesses. This approach will enhance the effectiveness of disease management measures. In this regard, wild *Vitis* species are an extremely useful pool of disease-responsive genes for environmental stresses. Some important genes that regulate stress response have been found in wild grapevines and these genes have been studied in Table 1. Currently, the CRISPR/Cas9 system has a wide range of genome editing capabilities, such as knock-in, knock-out, knockdown, and expression activation. The use of CRISPR/Cas9 systems for targeted mutagenesis shows promise in overcoming barriers to the development of superior quality and disease-resistant grape cultivars. Although there is still much to be accomplished in this field, recent developments indicate that advancement can enhance the *B. cinerea-*resistant or tolerant grape quality, yield, and secure storage of this commercially valuable and nutritious fruit by focusing on the most effective ways.

## Conclusion

Plant pathogens affect their growth, and the interaction between pathogen and host can lead to a weakening of the plant’s immune system. Plant diseases are caused by complex mechanisms that can be resolved by applying integrated defense techniques. Accelerated breeding programs are needed to combat gray mold infection of grape. Successful improvement is hampered by the many biological constraints of the grapevine as well as the genetic makeup of resistant traits. In this review, we have outlined the progress made in the characterization of the molecules involved in grapevine defense mechanisms and have shown that this knowledge is not complete and is only partially exploited in grapevine breeding. Significant advances have been made over the past decade in grape genome systems for susceptibility and resistance to *B. cinerea*. The research has produced extensive data that has uncovered novel information on the events and genes that regulate the development of *B. cinerea* infection in grapes. The primary goals of future grape breeding are to suppress infection and develop and introduce new varieties, maintain and improve nutritional quality, increase berry yields and restore plant traits inadvertently lost through artificial selection. Conventional breeding and hybridization are currently being replaced by more relevant new biotechnologies such as genome editing. As a result, the range of plant traits that can be achieved has expanded significantly and breeding efficiency has improved. In recent years, site-directed mutagenesis technologies such as ZFN, TALENs, and the CRISPR/Cas9 system have been used and have contributed significantly to enhancing resistance in fruits. In addition to laboratory research, special emphasis should be placed on studies that examine the interaction in the field and its response to environmental influences.

## Data Availability

All the data is presented in the main text file.
